# Glutathione *S*-Transferase Alpha 4 Promotes Proliferation and Chemoresistance in Colorectal Cancer Cells

**DOI:** 10.3389/fonc.2022.887127

**Published:** 2022-07-01

**Authors:** Zhanhu Zhang, Lili Xu, Lin Huang, Tianqi Li, Jane Y. Wang, Chunhua Ma, Xiaoyun Bian, Xiaoyan Ren, Haibo Li, Xingmin Wang

**Affiliations:** ^1^ Institute of Genetics and Reproductive Medicine, Affiliated Maternity and Child Healthcare Hospital of Nantong University, Nantong, China; ^2^ Department of Gastroenterology, Affiliated Maternity and Child Healthcare Hospital of Nantong University, Nantong, China; ^3^ Department of Internal Medicine, Washington University School of Medicine in St. Louis, St. Louis, MO, United States; ^4^ Department of Pathology, Affiliated Maternity and Child Healthcare Hospital of Nantong University, Nantong, China; ^5^ Department of Clinical Laboratory, Affiliated Maternity and Child Healthcare Hospital of Nantong University, Nantong, China

**Keywords:** glutathione *S*-transferase alpha 4, colorectal cancer, proliferation, reactive oxygen species, chemoresistance

## Abstract

Glutathione *S*-transferase alpha 4 (GSTA4) is a phase II detoxifying enzyme that is overexpressed in colorectal cancer (CRC) and regulated by the oncogenic transcription factor AP-1. However, the role of GSTA4 in these CRC cells remains unclear. In this study, we investigated the roles of GSTA4 in the CRC cells by inactivating GSTA4 in HCT116 human CRC cells (Defined as HCT116^ΔGSTA4^) using the CRISPR/Cas9 gene editing. Cell proliferation, clonogenicity, and susceptibility to chemotherapeutic drugs were analyzed *in vitro* and in a xenograft model. The results showed that loss of GSTA4 significantly decreased cell proliferation and clonogenicity, whereas it increased intracellular reactive oxygen species and cell susceptibility to 5-fluorouracil (5-FU) and oxaliplatin. Additionally, exposure of HCT116^ΔGSTA4^ cells to 5-FU increased the expression of γH2AX, a hallmark of double-stranded DNA breaks. In contrast, no remarkably increased γH2AX was noted in oxaliplatin-treated HCT116^ΔGSTA4^ cells compared with HCT116 cells. Moreover, loss of GSTA4 blocked the AKT and p38 MAPK pathways, leading to proliferative suppression. Finally, the xenograft model showed decreased tumor size for HCT116^ΔGSTA4^ cells compared with HCT116 cells, confirming *in vitro* findings. These findings suggest that GSTA4 is capable of promoting proliferation, tumorigenesis, and chemoresistance and is a potential target for CRC therapy.

## Introduction

Glutathione *S*-transferases (GSTs) are phase II detoxifying enzymes that are responsible for detoxifying xenobiotics and endogenous metabolites of oxidative stress (1). GSTs are also involved in cell signal transduction, post-translational modification, and resistance to chemotherapeutic drugs (2). In humans, at least 7 classes of GST exist prominently in the cytosol and, for certain classes, may be found in the mitochondria or membrane (3). Accumulating evidence has demonstrated that GSTs are associated with human cancers. Several classes of GSTs are highly expressed in human cancers, including colon, kidney, pancreatic, and liver cancers, implying cancer-promoting effects (4–7). Overexpression of GSTs can protect cancer cells against oxidative stress and/or promote cell proliferation through interactions with many growth-promoting molecules. For example, in a xenograft tumor model, deletion of GSTP1 significantly decreased tumor size *via* blocking the mitogen-activated protein kinase (MAPK) signaling pathway (8). This same GSTP1, on the other hand, can be a tumor suppressor in the *Apc* ^Min/+^ mouse model, in which the deletion of GSTP1 results in increased colon cancer incidence and tumor multiplicity (9). Additionally, epigenetic down-regulation of GSTP1 expression has been associated with increased susceptibility to prostate cancer (10, 11).

The human alpha class GST consists of 5 isozymes that are responsible for detoxifying a broad range of xenobiotics, namely, carcinogens, mutagens, chemotherapeutic drugs, steroids, and byproducts of oxidative stress (12). Some class A GSTs may also be associated with human cancers as tumor suppressors or tumor promoters. Recent studies have found that GSTA1 can suppress hepatocellular carcinoma progression and overexpression of GSTA1 is correlated with a better prognosis for patients (13). In contrast, GSTA2 promotes recurrence of hepatocellular carcinoma by regulating reactive oxygen species (ROS)-associated JNK and AKT signaling pathways (5). GSTA4 is a class A isoenzyme that can specifically detoxify 4-hydroxynonenal (4-HNE), a lipid peroxidation product of ω-6 polyunsaturated fatty acid. Single-nucleotide polymorphisms of the GSTA4 gene are associated with an increased risk of human nonmelanoma skin cancer, and inactivation of Gsta4 increases susceptibility to skin cancer in a murine model (14). Previous studies by our group have shown that GSTA4 is overexpressed in the biopsies of adenomas and invasive carcinomas from human beings and murine models (6). However, little is known whether this highly expressed GSTA4 contributes to the progression and/or chemoresistance of colorectal cancer (CRC).

Adjuvant chemotherapy has significant survival benefits for patients with late-stage CRC. However, chemoresistance often occurs during chemotherapy, leading to treatment failure (15, 16). Chemoresistance may be caused by decreased drug influx, increased drug efflux, and altered signaling pathways involved in cell proliferation and cell death. GSTs can metabolize chemotherapeutic drugs leading to chemoresistance. Recent studies have shown that overexpression of GSTP1 or GSTA1 is associated with cisplatin resistance in human lung, gastric, and ovarian cancers (17, 18). In contrast, hypermethylation of the GSTM1 gene reduces GSTM1 expression, which is associated with increased gemcitabine susceptibility in pancreatic cancer (19). 5-fluorouracil (5-FU) and oxaliplatin are two major chemotherapeutic drugs that are usually used in combination regimens such as FOLFOX (the combination of 5-FU, leucovorin, and oxaliplatin) for treating advanced CRC (16). However, acquired resistance to 5-FU and oxaliplatin often occurs during mid-treatment, which can significantly reduce survival benefits for CRC patients. Multiple mechanisms and many signaling molecules are involved in the development of chemoresistance against 5-FU and oxaliplatin. Of these, xenobiotic metabolizing enzymes such as GSTs, are important contributors to chemoresistance (20, 21). Overexpression of GSTA4 and GSTP1 is associated with the development of cisplatin resistance in human cancer cells of erythroleukemia, mammary, and ovary adenocarcinomas (22). Recent studies have shown that GSTA4 can reduce cisplatin-induced ototoxicity (23). Whether or not overexpression of GSTA4 contributes to chemoresistance in CRC is still unknown.

In this study, we inactivated GSTA4 in HCT116 human colon cancer cells and investigated the effect of GSTA4 on cancer cell proliferation and chemoresistance. We found that the inactivation of GSTA4 decreased cell proliferation and increased intracellular ROS. Additionally, the inactivation of GSTA4 significantly increased the susceptibility of HCT116 cells to the chemotherapeutic drugs 5-FU and oxaliplatin. Furthermore, inactivation of GSTA4 blocked the AKT and p38 MAPK pathways that are responsible for proliferative suppression. Finally, inactivation of GSTA4 inhibited xenograft tumor growth and increased susceptibility to 5-FU and oxaliplatin *in vivo*. These findings demonstrate that overexpression of GSTA4 promotes CRC cell proliferation, tumorigenesis, and chemoresistance, indicating that GST A4 blockade may lead to clinical benefits in CRC therapy.

## Materials and Methods

### Cell Culture

The HCT116 human colon cancer cell line was purchased from the National Collection of Authenticated Cell Cultures of China (Shanghai, China). HCT116 cells were cultured in McCoy’s 5A medium (Wisent, Nanjing, China) supplemented with 10% fetal bovine serum (Gibco, Carlsbad, CA), 100 μg/ml streptomycin, and 100 units/ml penicillin G in 5% CO_2_ at 37°C and subcultured by trypsinization when confluent.

### Inactivation of the GSTA4 Gene

**Figure 1:**
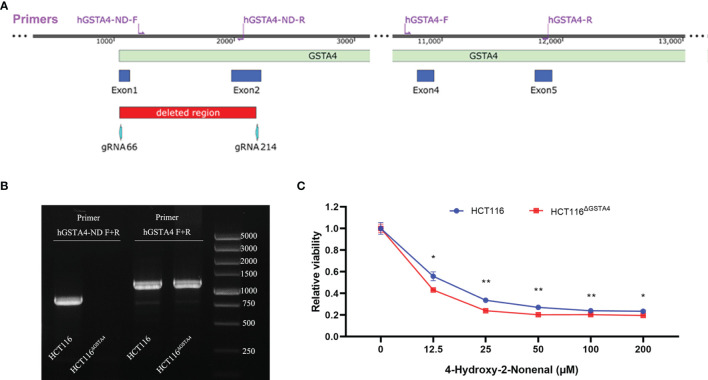
Deletion of GSTA4 using CRISPR/Cas9 gene editing. **(A)** The schematic diagram of the partial human GSTA4 gene and upstream sequence shows the positions of gRNAs, primers, and deleted fragments in the GSTA4 gene. **(B)** PCR photograph shows an 871 bp amplicon for HCT116 cells and negative amplification for HCT116^ΔGSTA4^ cells using primers hGSTA4-ND-F and hGSTA4-ND-R (Left lanes). A 1,189 bp fragment, as a control, is amplified for both HCT116 and HCT116^ΔGSTA4^ cells (Right lanes). **(C)** Inactivation of GSTA4 significantly increases 4-HNE-induced cell killing following a 48-hour exposure to various doses of 4-HNE. All data represent the mean ± SEM for three independent experiments. *P <0.05; **P <0.01.

Inactivation of the GSTA4 gene was carried out using the lentiCRISPRv2 system (Addgene, MA, USA) (24). Guide RNA (gRNA) sequences were designed to target the human GSTA4 gene using CRISPRdirect (https://crispr.dbcls.jp) (25). Selected gRNAs (**Supplementary Table S1**) were cloned into the pLentiCRISPRv2 vector and transformed into *E. coli* Stbl3 competent cells (TransGen Biotech, Beijing, China) as previously reported (24, 26). The positive transformants were screened by PCR and plasmids were extracted using a TaKaRa MiniBEST Plasmid Purification Kit (Takara, Dalian, China). To delete a large fragment in the GSTA4 gene, HCT116 cells were co-transfected with gRNA66 and gRNA214 (**Supplementary Table S1** and **Figure 1**) using Lipofectamine 3000 reagent (Invitrogen, Carlsbad, USA) according to the instructions of the manufacturer. Transfected cells were screened by 4 µg/ml puromycin followed by a maintenance concentration of 1 µg/ml puromycin. Clones surviving at 1 µg/ml puromycin were sub-cultured in a 24-well plate. DNA was extracted for mutant screening. Deletion of the GSTA4 gene fragment was detected by PCR amplification using specific primers (**Supplementary Table S1**) followed by Sanger sequencing.

### Cell Viability

Cell viability was determined using the CCK-8 assay kit (Beyotime Biotechnology, Shanghai, China). Cells were seeded in 96-well plates (5 × 10^3^ cells/well) and incubated overnight at 37°C. Following a 48-hour treatment with 5-FU or oxaliplatin, 10 μl of CCK-8 solution was added to each well and incubated at 37°C for 4 h. The absorbance was measured at 450 nm using a Varioskan LUX microplate reader (Thermo Fisher Scientific, MA, USA).

### Cell Proliferation Assay

Cell proliferation was determined using the BeyoClick™ EdU-555 kit (Beyotime Biotechnology) according to the instructions of the manufacturer. Briefly, cells were seeded in 6-well plates at a density of 2.5 × 10^5^ cells/well and incubated at 37°C overnight. Cells were treated with 5-FU or oxaliplatin for 48 h and incubated with 10 μM 5-ethynyl-2′-deoxyuridine (EdU) at 37°C for 2 h. Cells were then harvested by trypsinization, fixed with 4% paraformaldehyde at room temperature (RT) for 15 min, and permeabilized with 0.3% Triton X-100 in PBS at RT for 10 min. Cells were washed, stained with 500 μl of Click Additive Solution at RT for 30 min, and sorted on a DxFLEX flow cytometer (Beckman Coulter, Suzhou, China). EdU-positive cells were analyzed using CytExpert software.

### Clonogenic Assay

Cells were seeded into 6‐well plates at a density of 1,000 cells/well and cultured in 5% CO_2_ at 37°C for 10 days. The clones were fixed with methanol and stained with 0.1% crystal violet for 30 min. The number of clones was counted.

### Apoptosis Analysis

Apoptosis was analyzed using an Annexin-V/PI Staining kit (Vazyme, Nanjing, China) according to the instructions of the manufacturer. In brief, cells were harvested by trypsinization, washed with PBS, and counted. Cells (1 × 10^5^) were resuspended in 100 µl binding buffer and incubated with 5 µl Annexin V-FITC and 5 µl propidium iodide (PI) at RT for 10 min. Following staining, 400 µl binding buffer was added and cells were sorted on a DxFLEX flow cytometer (Beckman Coulter). The percentage of total apoptotic cells was calculated.

### Reactive Oxygen Species Analysis

ROS was detected using a ROS Assay Kit (Beyotime Biotechnology). Briefly, cells were treated with PBS, 5-FU, and oxaliplatin, respectively, for 48 h. Following treatment, 2’,7’-dichlorodihydrofluorescein diacetate (DCFH-DA) was added to a final concentration of 10 μM and incubated at 37°C for 30 min. Cells were harvested, washed with PBS, and re-seeded into a 96-well plate at a density of 2 × 10^4^ cells/well. The fluorescence intensity was measured at 525 nm using an excitation wavelength of 488 nm at the Varioskan LUX microplate reader (Thermo Fisher Scientific). The intensity of fluorescence was normalized to the fluorescence intensity of the PBS control.

### Western Blotting

Western blotting was performed as described previously (6). Cells were lysed using lysis buffer and the concentration was analyzed by the BCA assay (Beyotime Biotechnology). Whole-cell lysates were separated by SDS-PAGE, transferred to a PVDF membrane (Millipore, Shanghai, China), and blocked with 5% non-fat dry milk in Tris-buffered saline with 0.1% Tween 20 (TBST). The primary antibodies used in the study included anti-AKT (1:1,000, Affinity Biosciences, Changzhou, China), anti-p-AKT (1:1,000, Cell Signaling Technology, Shanghai, China), anti-p38 MAP kinase (1:1,000, Affinity Biosciences), anti-phosphorylated p38 (1:1,000, Cell Signaling Technology), and anti-β-actin (1:2,000, Sangon, Shanghai, China). HRP-conjugated species-specific secondary antibodies were purchased from Beyotime Biotechnology. Signals were generated by enhanced chemiluminescence (Biosharp, Hefei, China) and captured by the Odyssey Fc system (LI-COR Biosciences, Lincoln, NE).

### Xenograft Assay

The animal study was approved by the Institutional Animal Care and Use Committees at Nantong University. Male, 6‐week‐old specific pathogen-free BALB/cJGpt-*Foxn1^nu^*/Gpt nude mice were used for tumor xenograft. A total of 30 mice were randomly and equally divided into six groups, with 5 mice per group. HCT116 (3 groups) or GSTA4-deficient HCT116 (HCT116^ΔGSTA4^) cells (3 groups) were subcutaneously injected into the flanks of the mice (5 × 10^6^ cells/side) for xenograft tumor growth. One week after xenografting, mice were intraperitoneally injected with 5-FU (30 mg/kg), oxaliplatin (3 mg/kg), and saline as control, once a week for 4 weeks. Mouse body weight was recorded and tumor size was measured weekly. The tumor size was calculated as described previously (27). Mice were euthanized after 4-week treatment and tumors were removed for measuring size, weighing, and histology.

### Statistical Analysis

Data are expressed as means ± SEM from at least three independent experiments. Student’s *t*-test or two-way ANOVA was used for comparisons between groups using the GraphPad Prism 8 software (GraphPad Software, CA, USA). *P <*0.05 was considered statistically significant.

## Results

### Inactivation of GSTA4

To inactivate GSTA4 in HCT116 cells, we co-transfected HCT116 cells with pLentiCRISPR-gRNA66 and pLentiCRISPR-gRNA214 (**Supplementary Table S1**), which resulted in a 1,135 bp homozygous deletion across exons 1 and 2 in the GSTA4 gene (**Figure 1A**). Deletion of this fragment was confirmed by PCR amplification and sequence analysis (**Figure 1B** and **Supplementary Figure S1**). This GSTA4-deficient clone was named HCT116^ΔGSTA4^ and was used for the subsequent studies. Because GSTA4 is a detoxifying enzyme for 4-HNE, inactivation of GSTA4 in HCT116^ΔGSTA4^ was further confirmed by a decreased detoxifying capability for 4-HNE. The viability of HCT116^ΔGSTA4^ cells significantly decreased compared to the parental HCT116 cells when exposed to various concentrations of 4-HNE for 48 h (**Figure 1C**), indicating a reduced 4-HNE-detoxifying ability of HCT116^ΔGSTA4^ cells.

### Inactivation of GSTA4 Reduces Cell Proliferation

**Figure 2:**
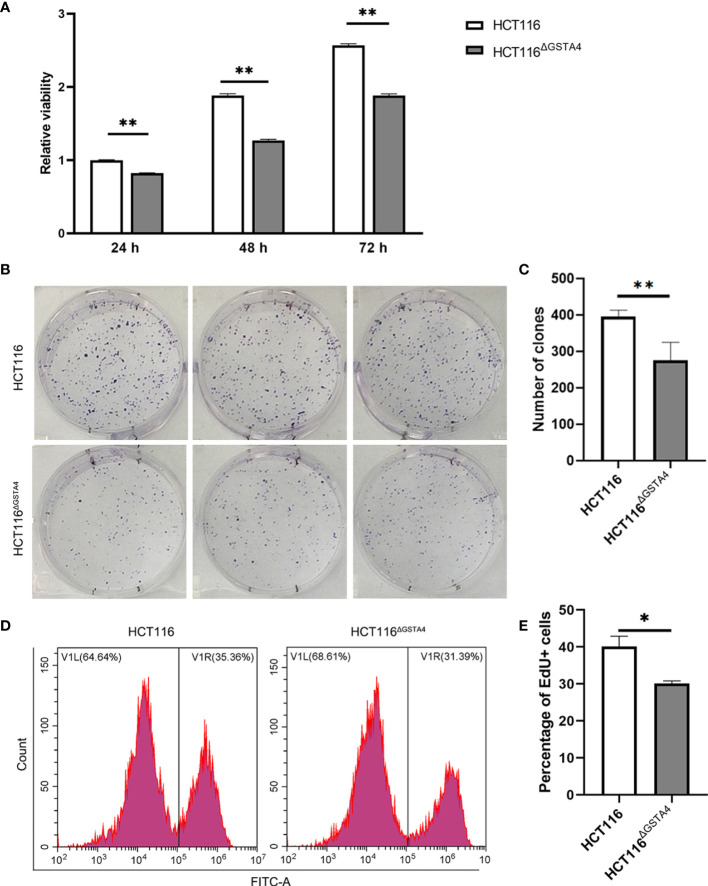
Inactivation of GSTA4 inhibits proliferation. **(A)** CCK8 assay shows remarkably decreased viability of HCT116^ΔGSTA4^ cells compared to the parental HCT116 cells. **(B)** Microphotographs of the clonogenic assay from triplicated wells. *Upper panels*, HCT116; *Lower panels*, HCT116^ΔGSTA4^. **(C)** Reduced number of clones is seen for HCT116^ΔGSTA4^ cells compared to HCT116 cells. **(D)** Representative histograms for FACS analysis of EdU incorporation assay. **(E)** The proportion of EdU-positive cells significantly decreases in HCT116^ΔGSTA4^ compared to HCT116 cells. All data represent the mean ± SEM for three independent experiments. *P <0.05; **P <0.01.

To investigate the effect of GSTA4 inactivation, we first determined the cell proliferation of HCT116^ΔGSTA4^ cells and compared them to the parental HCT116 cells. Inactivation of GSTA4 significantly reduced cell proliferation after culturing for 24, 48, and 72 h compared with HCT116 cells (**Figure 2A**). Furthermore, clonogenic assays demonstrated that the size and number of clones formed by HCT116^ΔGSTA4^ cells remarkably decreased compared with clones formed by HCT116 cells (**Figures 2B, C**). Additionally, decreased proliferation of HCT116^ΔGSTA4^ cells was confirmed by the EdU incorporation assay (**Figures 2D, E**). The fluorescence-activated cell sorting (FACS) analysis showed that the proportion of EdU-positive cells significantly decreased for HCT116^ΔGSTA4^ cells compared with HCT116 cells (32.6 ± 2.9% vs. 39.1 ± 4.6%, *P <*0.05). Finally, apoptosis was analyzed to determine whether the inactivation of GSTA4 induced apoptosis. FACS analysis showed that, when cells left untreated, there were no significant differences in the proportions of the early, late, and total apoptotic cells for HCT116^ΔGSTA4^ cells compared to those of HCT116 cells (**Supplementary Figure S2**, Ctrl). These results suggest that GSTA4 contributes to cancer cell proliferation while having no effect on apoptosis.

### Inactivation of GSTA4 Increases Response to Chemotherapeutic Agents

**Figure 3:**
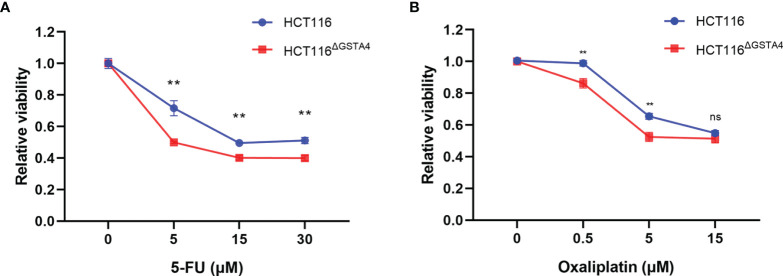
Inactivation of GSTA4 increases chemotherapeutic drug-induced cell killing. **(A, B)** CCK8 assay shows significantly decreased viability for HCT116^ΔGSTA4^ cells exposed to various doses of 5-FU **(A)** and oxaliplatin **(B)** compared to HCT116 cells. Data represent the mean ± SEM from three independent experiments. **P <0.01; ns, not significant.

GSTs, as phase II detoxifying enzymes, are associated with anti-cancer drug resistance (28). To investigate whether GSTA4 is involved in chemoresistance, we determined cell survival rates for HCT116 and HCT116^ΔGSTA4^ cells after exposure to 5-FU and oxaliplatin, two first-line chemotherapeutic agents for colorectal cancer. As shown in **Figure 3**, the survival rates significantly decreased for HCT116^ΔGSTA4^ cells compared with HCT116 cells following treatment with 5-FU at the doses of 5, 15, and 30 μM for 48 h. Similarly, significantly decreased survival rates were also noted for HCT116^ΔGSTA4^ cells exposed to oxaliplatin at the doses of 0.5 and 5 µM for 48 h. Notably, we were unable to find significantly increased proportions of early, late, and total apoptosis in HCT116 cells exposed to 5-FU (5 μM) and oxaliplatin (5 μM) compared with untreated controls (**Figure S2A–D**). Furthermore, inactivation of GSTA4 (HCT116^ΔGSTA4^) did not increase apoptosis compared with the parental HCT116 cells when treated with 5-FU (5 μM) and oxaliplatin (5 μM), suggesting that loss of GSTA4 augmented chemotherapeutic reduction of cell viability without affecting apoptosis.

**Figure 4:**
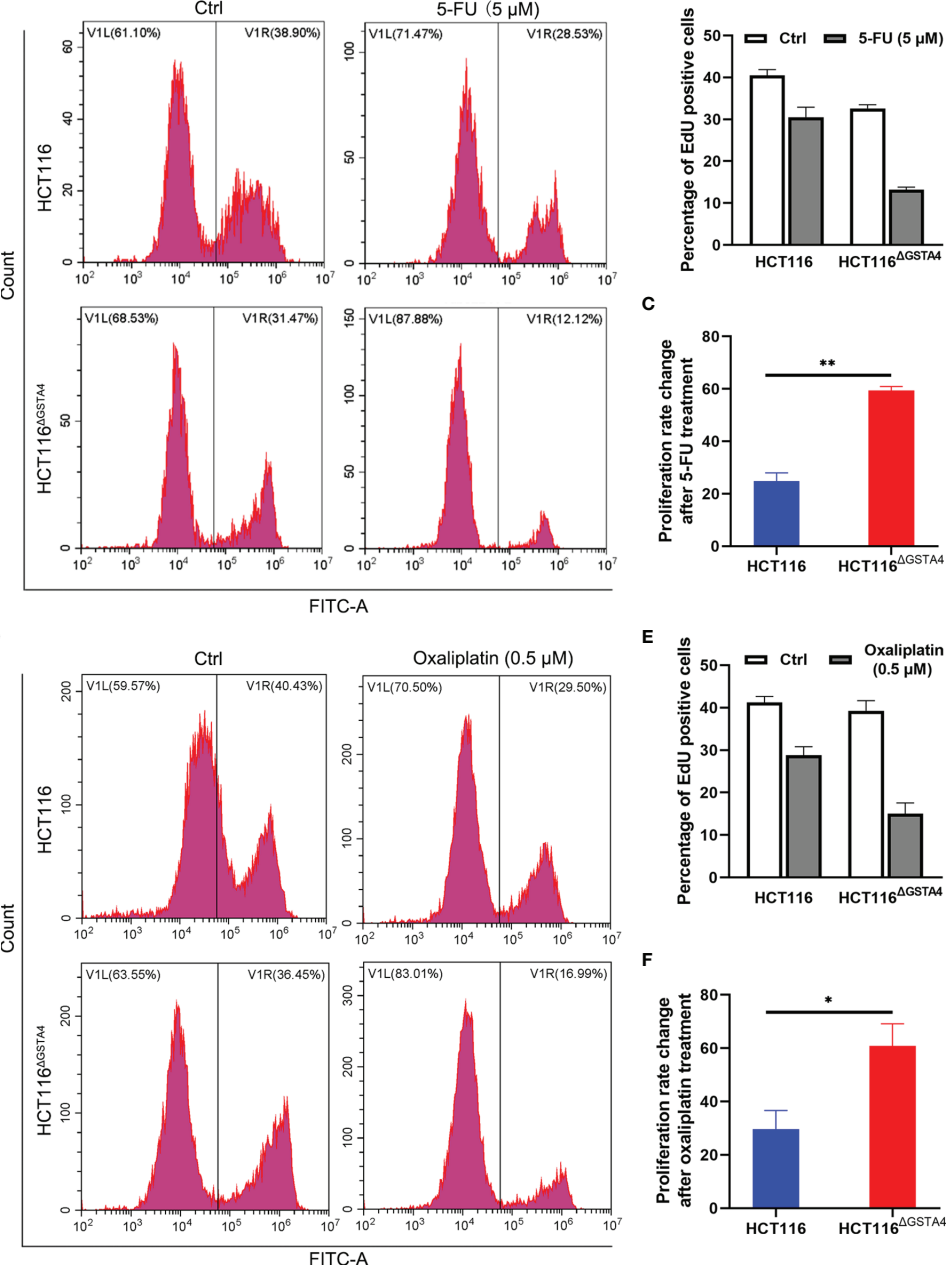
Inactivation of GSTA4 increases cell susceptibility to chemotherapeutics drugs. **(A)** Representative histograms for FACS analysis of the EdU incorporation assay following treatment with PBS or 5-FU. **(B)** The percentage of EdU-positive cells significantly decreases for HCT116^ΔGSTA4^ compared to HCT116 cells following 5-FU treatment. **(C)** Percent change of the EdU-positive cells for 5-FU-treated cells normalized to untreated cells. **(D)** Representative histograms for FACS analysis of EdU incorporation assay following treatment with PBS or oxaliplatin. **(E)** The percentage of EdU-positive cells significantly decreases for HCT116^ΔGSTA4^ compared to HCT116 cells following oxaliplatin treatment. **(F)** Percent change of EdU-positive cells for oxaliplatin-treated cells normalized to untreated cells. All data represent the mean ± SEM for three independent experiments. *P <0.05 and **P <0.01.

We next examined cell proliferation using the EdU incorporation assay following treatment with 5-FU and oxaliplatin. In comparison with untreated controls, the proportion of EdU-positive cells decreased for both HCT116 and HCT116^ΔGSTA4^ cells after treatment with 5-FU and oxaliplatin. Treatment with 5-FU (5 µM) for 48 h resulted in a 56.28 ± 4.93% decreased proportion of EdU-positive cells (from 32.59 ± 1.56% to 13.24 ± 0.98%, *P <*0.01) for HCT116^ΔGSTA4^ cells compared to a 19.22 ± 6.82% decrease (from 40.46 ± 2.40% to 30.53 ± 4.08%, *P <*0.05) for HCT116 cells (**Figures 4A–C**). Comparably, treatment with oxaliplatin (0.5 µM) for 48 h also resulted in a 56.77 ± 17.65% decreased proportion of EdU-positive cells (from 33.63 ± 4.04% to 10.01 ± 8.87%, *P <*0.01) for HCT116^ΔGSTA4^ cells compared to a 11.77 ± 9.79% decrease (from 38.40 ± 5.39% to 18.86 ± 14.21%, *P <*0.05) for HCT116 cells (**Figures 4D–F**). These results indicate that inactivation of GSTA4 increases the sensitivity of colorectal cancer cells to chemotherapeutic agents *via* inhibition of proliferation.

### Inactivation of GSTA4 Promotes ROS Production and DNA Damage

**Figure 5:**
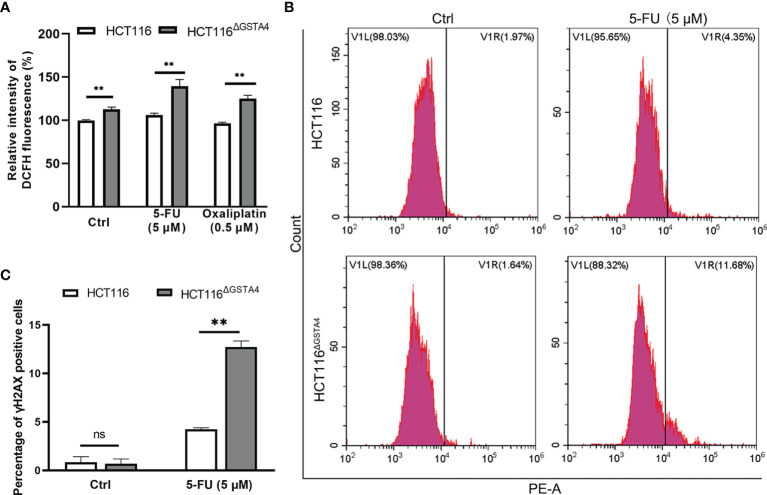
Inactivation of GSTA4 promotes ROS production and DNA damage. **(A)** ROS production significantly increases for HCT116^ΔGSTA4^ compared to HCT116 cells exposed to PBS, 5-FU, and oxaliplatin, respectively. **(B)** Representative histograms of FACS analysis for γH2AX. **(C)** No remarkable difference is noted in the percentage of γH2AX for untreated HCT116^ΔGSTA4^ and HCT116 cells. The percentage of γH2AX significantly increases for HCT116^ΔGSTA4^ compared to HCT116 cells following treatment with 5-FU. All data represent the mean ± SEM for three independent experiments. **P <0.01; ns, not significant.

Many chemotherapeutic agents act as DNA damage inducers, leading to cancer cell death. Inactivation of GSTs often induces ROS production that causes DNA damage (29). However, chemotherapeutic agents may also modulate GST expression and ROS production (5, 30). Therefore, we measured ROS production in HCT116^ΔGSTA4^ cells compared with that in HCT116 cells and found that inactivation of GSTA4 increased ROS production in untreated HCT116^ΔGSTA4^ cells in contrast to HCT116 cells. Furthermore, ROS production also increased significantly in HCT116^ΔGSTA4^ cells treated with 5-FU and oxaliplatin compared with HCT116 cells treated with the same drugs (**Figure 5A**). To determine whether the inactivation of GSTA4 promotes DNA damage, we analyzed phosphorylated H2AX (γH2AX), a hallmark of double-stranded DNA breaks, by FACS analysis following treatment with 5-FU and oxaliplatin. No significant change was seen in the proportion of γH2AX-positive cells between untreated HCT116^ΔGSTA4^ and HCT116 cells (0.7 ± 0.82% vs 0.84 ± 0.98%, *P* = 0.859), suggesting that loss of GSTA4 has no effect on DNA damage in unstressed cells. Following a 48-hour treatment with 5-FU, the proportion of γH2AX-positive cells increased to 12.73 ± 1.06% and 4.23 ± 0.28% for HCT116^ΔGSTA4^ and HCT116 cells, respectively (**Figures 5B, C**, *P <*0.01). In contrast, although the proportion of γH2AX-positive cells increased for both HCT116^ΔGSTA4^ (1.67 ± 2.42%) and HCT116 cells (3.69 ± 6.00%) after treatment with oxaliplatin for 48 h compared with untreated controls, no significant difference in the proportion of γH2AX-positive cells was seen between HCT116^ΔGSTA4^ and HCT116 cells (**Supplementary Figures 3A, B**, *P* = 0.618), indicating that the inactivation of GSTA4 has limited effect on low-dose oxaliplatin-induced crosslinking of DNA.

### Inactivation of GSTA4 Reduces Proliferation *via* Blocking AKT and p38 MAPK Signaling Pathways

**Figure 6:**
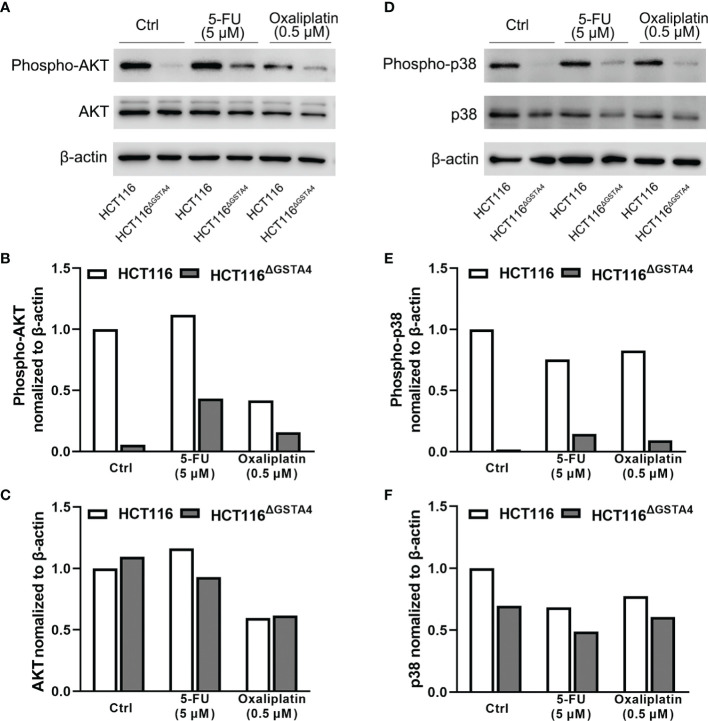
Inactivation of GSTA4 blocks AKT and MAPK signaling pathways. **(A–C)** Western blots show remarkable decrease of p-AKT in HCT116^ΔGSTA4^ compared to HCT116 cells treated with or without 5-FU or oxaliplatin after normalizing to β-actin. **(D–F)** Remarkably decreased p-p38 is seen in HCT116^ΔGSTA4^ compared to HCT116 cells treated with or without chemotherapeutic agents after normalizing to β-actin.

To further investigate the mechanisms by which inactivation of GSTA4 inhibits proliferation, we examined proliferative signaling pathways including phosphoinositide 3 kinase (PI3K) and MAPK. As shown in **Figure 6**, no remarkable change was noted in the expression of AKT (a downstream kinase in the PI3K signaling pathway) and p38 MAPK. However, phosphorylated AKT (p-AKT) and phosphorylated p38 (p-p38), the activated forms of AKT and p38, remarkably decreased in HCT116^ΔGSTA4^ cells compared with HCT116 cells, indicating inhibition of AKT and p38 signaling pathways. Additionally, treatment of HCT116 cells with 5-FU and oxaliplatin slightly decreased p-AKT and p-p38 pathways in HCT116 cells while being prone to increasing p-AKT and p-p38 in HCT116^ΔGSTA4^ cells. These results suggest that inactivation of GSTA4 reduces proliferation by blocking PI3K/AKT and p38 MAPK signaling pathways and that activation of AKT and p38 pathways in HCT116 CRC cells occurs in a GSTA4-dependent manner.

### Inactivation of GSTA4 Inhibits Xenograft Tumorigenesis

**Figure 7:**
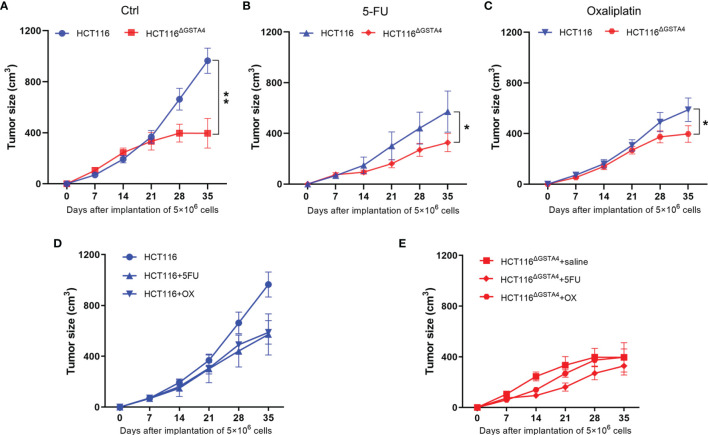
Inactivation of GSTA4 inhibits xenograft tumor growth and increases tumor susceptibility to chemotherapeutic drugs *in vivo*. **(A–C)** Significantly decreased tumor size is noted for HCT116^ΔGSTA4^-derived xenografts compared to HCT116-derived xenografts when exposed to saline **(A)**, 5-FU **(B)**, and oxaliplatin **(C)**, respectively. **(D)** Xenograft tumor size for HCT116 cells exposed to saline, 5-FU, and oxaliplatin. Both 5-FU and oxaliplatin reduce tumor size compared to saline control (*P <*0.05 for both comparisons). No difference is seen between 5-FU and oxaliplatin (*P* = 0.75). **(E)** Xenograft tumor size for HCT116^ΔGSTA4^ cells exposed to saline, 5-FU, and oxaliplatin. 5-FU (*P <*0.05), not oxaliplatin (*P* = 0.22), reduces tumor size compared to saline control. Data are expressed as means ± SEM. Two-way ANOVA is used for all comparisons. *P <0.05 and **P <0.01.

Finally, we investigated the effect of GSTA4-deficiency on tumorigenesis using a xenograft tumor model. We injected nude mice with 5 × 10^6^ cells per side of the flank and treated them with saline, 5-FU, and oxaliplatin, respectively. For saline-treated mice, the average tumor size significantly decreased for HCT116^ΔGSTA4^-derived xenografts compared with HCT116-derived xenografts (*P <*0.001), indicating reduced tumor growth for GSTA4-deficient HCT116 cells (**Figure 7A**). For mice treated with 5-FU and oxaliplatin, the average tumor size also significantly decreased for xenografts derived from HCT116^ΔGSTA4^ cells compared with HCT116 cells (**Figures 7B, C**, *P <*0.05 for both 5-FU and oxaliplatin groups). Of note, both 5-FU and oxaliplatin reduced tumor size in HCT116-derived xenografts compared with saline control. However, no significant difference was found between 5-FU and oxaliplatin (**Figure 7D**, *P* = 0.75). In contrast, for HCT116^ΔGSTA4^-derived xenografts, 5-FU, but not oxaliplatin, remarkably reduced tumor size compared to saline control (**Figure 7E**, *P <*0.01 and *P* = 0.22 for 5-FU and oxaliplatin, respectively). This is consistent with the findings that 5-FU, but not oxaliplatin, at the tested doses, induced more γH2AX foci in HCT116^ΔGSTA4^ cells. These results indicate that GSTA4 promotes *in vivo* tumorigenesis and contributes to chemoresistance.

## Discussion

GSTA4 is overexpressed in the colon biopsies of human colorectal cancer and the bacteria-induced murine CRC model through activating oncogenic transcription factor activator protein 1 (AP-1) (6). The impact of the overexpressed GSTA4 on the CRC cells is, however, unclear. In this study, we investigated the role of GSTA4 in CRC cells by deleting GSTA4 in HCT116 human colon cancer cells. Our results show that inactivation of GSTA4 in HCT116 cells reduces cell proliferation and increases chemotherapeutic drug sensitivity *in vitro* and in a xenograft tumor model. Inactivation of GSTA4 increases intracellular ROS production and blocks proliferative signaling pathways, including AKT and p38 MAPK. These results show that GSTA4 activation by AP-1 in CRC promotes tumor growth *via* activation of proliferative signaling pathways.

Several GSTs regulate proliferation, tumorigenesis, tumor progression, and metastasis (5, 31, 32). These GSTs can directly interact with various signaling molecules to regulate proliferation. For example, GSTO1-1 interacts with AKT and MEK1/2, activating AKT signaling in human neuroblastoma cells (33). Additionally, GSTP1 can regulate cell cycle progression and proliferation through direct interaction with JNK and TNF receptor-associated factor 2 (TRAF2) (32, 34). Silencing of GSTP1 in pancreatic ductal adenocarcinoma cells can affect both proliferation and apoptosis (7). While little is known about the role of GSTA4 in cancer, particularly in colorectal cancer, previous studies have demonstrated that transfection of primary cell lines with human GSTA4-expressing plasmids causes cellular transformation and immortalization *via* upregulation of transforming growth factor, cyclin-dependent kinase 2, and protein kinase C beta II and downregulation of p53 (35). Our previous results showed that colorectal cancer cells expressed GSTA4 (6) and we show in the current study that inactivation of GSTA4 in HCT116 cells decreases proliferation *via* inhibition of PI3K/MAPK pathways, supporting the tumor promotive role of GSTA4. Nonetheless, the mechanisms of how GSTA4 regulates AKT and p38 activation are still unknown. One potential process is that GSTA4, like other GSTs, can directly interact with AKT and p38. Further investigation is needed to confirm this hypothesis in HCT116^ΔGSTA4^ and HCT116 cells in which GSTA4 is silenced by *GSTA4*-specific siRNA or shRNA.

4-HNE is a byproduct of lipid peroxidation that can alter protein functions by forming 4-HNE-protein adducts, induce DNA damage, and act as a signaling inducer (36, 37). 4-HNE also induces apoptosis and inhibits proliferation by activating p21 and c-Myc in CRC cells (38). Because GSTA4 specifically conjugates glutathione to 4-HNE and detoxifies 4-HNE (37), we expected that inactivation of GSTA4 was capable of increasing 4-HNE-induced cell death, presumably *via* apoptosis caused by accumulating 4-HNE. However, this study shows that inactivation of GSTA4 has no effect on apoptosis in HCT116 cells. The survival rate indeed decreased significantly for 4-HNE-treated HCT116^ΔGSTA4^ cells compared with the parental HCT116 cells. Nearly 20% of cells, however, still survived after 200 μM 4-HNE treatment, indicating that alternative 4-HNE-detoxifying mechanisms exist besides the GSTA4-mediated phase II detoxifying pathway. These detoxifying mechanisms may be phase I detoxifying enzymes such as aldehyde dehydrogenases, alcohol dehydrogenases, aldo-keto reductase, and other enzymes (39). Whether or not these enzymes could be compensatively up-regulated by 4-HNE in GSTA4-deficient cells is unclear. This is an interesting topic that may merit further investigation but is beyond the scope of this study.

The tumor microenvironment is enriched with ROS that have multifaceted roles in cancer cell proliferation, apoptosis, angiogenesis, and metastasis (29). GSTs, as anti-oxidant enzymes, can eliminate ROS and protect cells against ROS-induced apoptosis (5, 8). As such, we speculate that the deletion of GSTA4 in HCT116 cells also increases intracellular ROS. The results from this study show that inactivation of GSTA4, as expected, increases ROS for untreated cells and cells treated with 5-FU and oxaliplatin. Severely increased ROS can induce apoptosis, but slightly increased ROS, on the other hand, may promote proliferation and angiogenesis (40). ROS regulates proliferation and apoptosis through various signaling pathways, including AKT and MAPK (29, 40). ROS can also promote CRC cell proliferation through the NOX1 pathway (41). Notably, no increased apoptosis was seen in HCT116^ΔGSTA4^ cells regardless of increased ROS, implying that inactivation of GSTA4 mildly increases ROS that might promote proliferation. Interestingly, we have not seen increased proliferation or apoptosis despite increased ROS in HCT116^ΔGSTA4^ cells. Therefore, the slightly increased ROS may not be associated with the blockage of proliferation in HCT116^ΔGSTA4^ cells, showing that GSTA4 regulates proliferation by direct interaction with growth signaling molecules such as AKT and p38.

Oxaliplatin and 5-FU are commonly used for the treatment of early-stage CRC as adjuvant chemotherapy and the first-line chemotherapy for the metastatic CRC (42). Although both 5-FU and oxaliplatin can cause DNA damage and, eventually, result in cancer cell death, their mechanisms of action are different. 5-FU is an anti-metabolite agent that inhibits thymidylate synthase and incorporates its metabolites into DNA and RNA, leading to DNA damage and cancer cell death (43). Oxaliplatin, on the other hand, can form oxaliplatin-DNA adducts and crosslinks, leading to apoptosis (44). Although both 5-FU and oxaliplatin can activate γH2AX (45, 46), we found increased γH2AX in HCT116^ΔGSTA4^ cells treated with 5-FU but not with oxaliplatin. This may be due to the extremely low dosage of oxaliplatin (0.5 µM) that we have tested in these experiments. Notably, previous studies have demonstrated that exposure of SW480 CRC cells to oxaliplatin at a dose of 10 µM, which is 20-fold of the dose that we used to treat HCT116 cells in this study, induced a remarkably low proportion of γH2AX-positive cells (47). In congruence with the results of γH2AX expression, we noted relatively low proportions of apoptotic cells in both HCT116 and HCT116^ΔGSTA4^ cells treated with 5-FU and oxaliplatin, which is similar to the rates of apoptosis in HCT116 cells treated with these drugs as reported by other investigators (48). Finally, the different mechanisms of action for 5-FU and oxaliplatin may explain why 5-FU, but not oxaliplatin, increases γH2AX caused by the inactivation of GSTA4. Concomitantly, no significantly decreased tumor size was noted for HCT116^ΔGSTA4^-derived xenografts treated with oxaliplatin compared to the untreated group (**Figure 7E**). This was due to a significant decrease in tumor size in the control group. Previous studies have found that overexpression of GSTA2 protects colon cancer cells against crosslinking agent-induced DNA damage (49). Whether or not inactivation of GSTA4 increases oxaliplatin-induced crosslinks is still unknown. Nevertheless, inactivation of GSTA4 remarkably increases cell susceptibility to chemotherapeutic agents *in vitro* and in the xenograft mouse model.

In conclusion, inactivation of GSTA4 decreases CRC cell proliferation by blocking AKT and p38 MAPK signaling pathways. Additionally, inactivation of GSTA4 increases intracellular ROS and cancer cell susceptibility to the chemotherapeutic agents, 5-FU and oxaliplatin. These results suggest that activation of GSTA4 in CRC cells promotes tumorigenesis and contributes to chemoresistance, and that GSTA4 is a potential therapeutic target for treating CRC.

## Data Availability Statement

The original contributions presented in the study are included in the article/**Supplementary Material**. Further inquiries can be directed to the corresponding author.

## Ethics Statement

The animal study was reviewed and approved by the Institutional Animal Care and Use Committees at Nantong University.

## Author Contributions

ZZ performed research, analyzed data, prepared figures, and drafted the manuscript. LX and CM performed gene manipulation, created the GSTA4-deficient cell line, and analyzed data. LH and TL performed research and analyzed data. JW interpreted data, edited and critically reviewed the manuscript. XR was responsible for pathological evaluation. XB and HL analyzed data and interpreted results. XW designed the study, analyzed data, wrote the manuscript, revised figures, and supervised the research. All authors listed have made a substantial, direct, and intellectual contribution to the work and approved it for publication.

## Funding

This study was supported by the Research Program from Jiangsu Commission of Health (H2018109 to XW), the National Natural Science Foundation of China (81972783 to XW), the Jiangsu Distinguished Medical Experts Program (to XW), the Jiangsu Health Innovation Team Program (to XM), the Jiangsu 333 Talent Project (to HL), and the Nantong Commission of Health (MA2021029 to LX).

## Conflict of Interest

The authors declare that the research was conducted in the absence of any commercial or financial relationships that could be construed as a potential conflict of interest.

## Publisher’s Note

All claims expressed in this article are solely those of the authors and do not necessarily represent those of their affiliated organizations, or those of the publisher, the editors and the reviewers. Any product that may be evaluated in this article, or claim that may be made by its manufacturer, is not guaranteed or endorsed by the publisher.

## References

[1] Hayes JD Flanagan JU Jowsey IR. Glutathione Transferases. Annu Rev Pharmacol Toxicol (2005) 45:51–88. doi: 10.1146/annurev.pharmtox.45.120403.095857 15822171

[2] Singh RR Reindl KM. Glutathione S-Transferases in Cancer. Antioxidants (2021) 10(5):1–25. doi: 10.3390/antiox10050701 PMC8146591 33946704

[3] Chatterjee A Gupta S. The Multifaceted Role of Glutathione S-Transferases in Cancer. Cancer Lett (2018) 433:33–42. doi: 10.1016/j.canlet.2018.06.028 29959055

[4] Searchfield L Price SA Betton G Jasani B Riccardi D Griffiths DF. Glutathione S-Transferases as Molecular Markers of Tumour Progression and Prognosis in Renal Cell Carcinoma. Histopathology (2011) 58(2):180–90. doi: 10.1111/j.1365-2559.2010.03733.x 21255063

[5] Ng KT Yeung OW Lam YF Liu J Liu H Pang L. Glutathione S-Transferase A2 Promotes Hepatocellular Carcinoma Recurrence After Liver Transplantation Through Modulating Reactive Oxygen Species Metabolism. Cell Death Discovery (2021) 7(1):188. doi: 10.1038/s41420-021-00569-y 34290233 PMC8295304

[6] Yang Y Huycke MM Herman TS Wang X. Glutathione S-Transferase Alpha 4 Induction by Activator Protein 1 in Colorectal Cancer. Oncogene (2016) 35(44):5795–806. doi: 10.1038/onc.2016.113 PMC9619338 27065323

[7] Singh RR Mohammad J Orr M Reindl KM. Glutathione S-Transferase Pi-1 Knockdown Reduces Pancreatic Ductal Adenocarcinoma Growth by Activating Oxidative Stress Response Pathways. Cancers (2020) 12(6):1–22. doi: 10.3390/cancers12061501 PMC7352757 32526885

[8] Dang DT Chen F Kohli M Rago C Cummins JM Dang LH. Glutathione S-Transferase Pi1 Promotes Tumorigenicity in Hct116 Human Colon Cancer Cells. Cancer Res (2005) 65(20):9485–94. doi: 10.1158/0008-5472.CAN-05-1930 16230413

[9] Ritchie KJ Walsh S Sansom OJ Henderson CJ Wolf CR. Markedly Enhanced Colon Tumorigenesis in Apc(Min) Mice Lacking Glutathione S-Transferase Pi. Proc Natl Acad Sci U S A (2009) 106(49):20859–64. doi: 10.1073/pnas.0911351106 PMC2777186 19915149

[10] Martignano F Gurioli G Salvi S Calistri D Costantini M Gunelli R. Gstp1 Methylation and Protein Expression in Prostate Cancer: Diagnostic Implications. Dis Markers (2016) 2016:4358292. doi: 10.1155/2016/4358292 27594734 PMC4995330

[11] Yamoah K Johnson MH Choeurng V Faisal FA Yousefi K Haddad Z. Novel Biomarker Signature That May Predict Aggressive Disease in African American Men With Prostate Cancer. J Clin Oncol (2015) 33(25):2789–96. doi: 10.1200/jco.2014.59.8912 PMC4550692 26195723

[12] Coles BF Kadlubar FF. Human Alpha Class Glutathione S-Transferases: Genetic Polymorphism, Expression, and Susceptibility to Disease. Methods Enzymol (2005) 401:9–42. doi: 10.1016/S0076-6879(05)01002-5 16399377

[13] Liu X Sui X Zhang C Wei K Bao Y Xiong J. Glutathione S-Transferase A1 Suppresses Tumor Progression and Indicates Better Prognosis of Human Primary Hepatocellular Carcinoma. J Cancer (2020) 11(1):83–91. doi: 10.7150/jca.36495 31892975 PMC6930411

[14] Abel EL Angel JM Riggs PK Langfield L Lo HH Person MD. Evidence That Gsta4 Modifies Susceptibility to Skin Tumor Development in Mice and Humans. J Natl Cancer Inst (2010) 102(21):1663–75. doi: 10.1093/jnci/djq392 PMC2970579 20966433

[15] Ortíz R Quiñonero F García-Pinel B Fuel M Mesas C Cabeza L. Nanomedicine to Overcome Multidrug Resistance Mechanisms in Colon and Pancreatic Cancer: Recent Progress. Cancers (2021) 13(9):1–27. doi: 10.3390/cancers13092058 PMC8123136 33923200

[16] Carrato A. Adjuvant Treatment of Colorectal Cancer. Gastrointest Cancer Res (2008) 2(4 Suppl):S42–6. PMC2661542 19343149

[17] Li J Ye T Liu Y Kong L Sun Z Liu D. Transcriptional Activation of Gstp1 by Mek/Erk Signaling Confers Chemo-Resistance to Cisplatin in Lung Cancer Stem Cells. Front Oncol (2019) 9:476(476). doi: 10.3389/fonc.2019.00476 31263672 PMC6584806

[18] Zou M Hu X Xu B Tong T Jing Y Xi L. Glutathione S−Transferase Isozyme Alpha 1 Is Predominantly Involved in the Cisplatin Resistance of Common Types of Solid Cancer. Oncol Rep (2019) 41(2):989–98. doi: 10.3892/or.2018.6861 30431119

[19] Tan AC Jimeno A Lin SH Wheelhouse J Chan F Solomon A. Characterizing DNA Methylation Patterns in Pancreatic Cancer Genome. Mol Oncol (2009) 3(5-6):425–38. doi: 10.1016/j.molonc.2009.03.004 PMC5527529 19497796

[20] Noordhuis P Laan AC van de Born K Honeywell RJ Peters GJ. Coexisting Molecular Determinants of Acquired Oxaliplatin Resistance in Human Colorectal and Ovarian Cancer Cell Lines. Int J Mol Sci (2019) 20(15):1–18. doi: 10.3390/ijms20153619 PMC6696456 31344863

[21] Azwar S Seow HF Abdullah M Faisal Jabar M Mohtarrudin N. Recent Updates on Mechanisms of Resistance to 5-Fluorouracil and Reversal Strategies in Colon Cancer Treatment. Biol (Basel) (2021) 10(9):1–34. doi: 10.3390/biology10090854 PMC8466833 34571731

[22] Kalinina EV Berozov TT Shtil AA Chernov NN Glasunova VA Novichkova MD. Expression of Genes of Glutathione Transferase Isoforms Gstp1-1, Gsta4-4, and Gstk1-1 in Tumor Cells During the Formation of Drug Resistance to Cisplatin. Bull Exp Biol Med (2012) 154:64–7. doi: 10.1007/s10517-012-1876-4 23330092

[23] Park HJ Kim MJ Rothenberger C Kumar A Sampson EM Ding D. Gsta4 Mediates Reduction of Cisplatin Ototoxicity in Female Mice. Nat Commun (2019) 10(1):4150. doi: 10.1038/s41467-019-12073-0 31515474 PMC6742643

[24] Sanjana NE Shalem O Zhang F. Improved Vectors and Genome-Wide Libraries for Crispr Screening. Nat Methods (2014) 11(8):783–4. doi: 10.1038/nmeth.3047 PMC4486245 25075903

[25] Naito Y Hino K Bono H Ui-Tei K. Crisprdirect: Software for Designing Crispr/Cas Guide Rna With Reduced Off-Target Sites. Bioinformatics (2015) 31(7):1120–3. doi: 10.1093/bioinformatics/btu743 PMC4382898 25414360

[26] Shalem O Sanjana NE Hartenian E Shi X Scott DA Mikkelson T. Genome-Scale Crispr-Cas9 Knockout Screening in Human Cells. Science (2014) 343(6166):84–7. doi: 10.1126/science.1247005 PMC4089965 24336571

[27] Faustino-Rocha A Oliveira PA Pinho-Oliveira J Teixeira-Guedes C Soares-Maia R da Costa RG. Estimation of Rat Mammary Tumor Volume Using Caliper and Ultrasonography Measurements. Lab Anim (2013) 42(6):217–24. doi: 10.1038/laban.254 23689461

[28] Townsend DM Tew KD. The Role of Glutathione-S-Transferase in Anti-Cancer Drug Resistance. Oncogene (2003) 22(47):7369–75. doi: 10.1038/sj.onc.1206940 PMC6361125 14576844

[29] Sorolla MA Hidalgo I Sorolla A Montal R Pallisé O Salud A. Microenvironmental Reactive Oxygen Species in Colorectal Cancer: Involved Processes and Therapeutic Opportunities. Cancers (2021) 13(20):1–23. doi: 10.3390/cancers13205037 PMC8534037 34680186

[30] Aybek H Temel Y Ahmed BM Ağca CA Çiftci M. Deciphering of the Effect of Chemotherapeutic Agents on Human Glutathione S-Transferase Enzyme and Mcf-7 Cell Line. Protein Pept Lett (2020) 27(9):888–94. doi: 10.2174/0929866527666200413101017 32282293

[31] Liu H Yang Z Zang L Wang G Zhou S Jin G. Downregulation of Glutathione S-Transferase A1 Suppressed Tumor Growth and Induced Cell Apoptosis in A549 Cell Line. Oncol Lett (2018) 16(1):467–74. doi: 10.3892/ol.2018.8608 PMC6006369 29928434

[32] Gate L Majumdar RS Lunk A Tew KD. Increased Myeloproliferation in Glutathione S-Transferase Pi-Deficient Mice Is Associated With a Deregulation of Jnk and Janus Kinase/Stat Pathways. J Biol Chem (2004) 279(10):8608–16. doi: 10.1074/jbc.M308613200 14684749

[33] Saisawang C Wongsantichon J Robinson RC Ketterman AJ. Glutathione Transferase Omega 1-1 (Gsto1-1) Modulates Akt and Mek1/2 Signaling in Human Neuroblastoma Cell Sh-Sy5y. Proteins (2019) 87(7):588–95. doi: 10.1002/prot.25683 30874320

[34] De Luca A Mei G Rosato N Nicolai E Federici L Palumbo C. The Fine-Tuning of Traf2-Gstp1-1 Interaction: Effect of Ligand Binding and in Situ Detection of the Complex. Cell Death Dis (2014) 5(1):e1015. doi: 10.1038/cddis.2013.529 24457959 PMC4040697

[35] Sharma R Brown D Awasthi S Yang Y Sharma A Patrick B. Transfection With 4-Hydroxynonenal-Metabolizing Glutathione *S-*Transferase Isozymes Leads to Phenotypic Transformation and Immortalization of Adherent Cells. Eur J Biochem (2004) 271:1690–701. doi: 10.1111/j.1432-1033.2004.04067.x. 15096208

[36] Wang X Yang Y Moore DR Nimmo SL Lightfoot SA Huycke MM. 4-Hydroxy-2-Nonenal Mediates Genotoxicity and Bystander Effects Caused by *Enterococcus faecalis-*Infected Macrophages. Gastroenterology (2012) 142:543–51. doi: 10.1053/j.gastro.2011.11.020 PMC3371374 22108198

[37] Guéraud F. 4-Hydroxynonenal Metabolites and Adducts in Pre-Carcinogenic Conditions and Cancer. Free Radic Biol Med (2017) 111:196–208. doi: 10.1016/j.freeradbiomed.2016.12.025 28065782

[38] Cerbone A Toaldo C Laurora S Briatore F Pizzimenti S Dianzani MU. 4-Hydroxynonenal and Ppargamma Ligands Affect Proliferation, Differentiation, and Apoptosis in Colon Cancer Cells. Free Radic Biol Med (2007) 42(11):1661–70. doi: 10.1016/j.freeradbiomed.2007.02.009 17462534

[39] Mol M Regazzoni L Altomare A Degani G Carini M Vistoli G. Enzymatic and Non-Enzymatic Detoxification of 4-Hydroxynonenal: Methodological Aspects and Biological Consequences. Free Radic Biol Med (2017) 111:328–44. doi: 10.1016/j.freeradbiomed.2017.01.036 28161307

[40] Sun Y Lu Y Saredy J Wang X Drummer Iv C Shao Y. Ros Systems Are a New Integrated Network for Sensing Homeostasis and Alarming Stresses in Organelle Metabolic Processes. Redox Biol (2020) 37:101696. doi: 10.1016/j.redox.2020.101696 32950427 PMC7767745

[41] Juhasz A Markel S Gaur S Liu H Lu J Jiang G. Nadph Oxidase 1 Supports Proliferation of Colon Cancer Cells by Modulating Reactive Oxygen Species-Dependent Signal Transduction. J Biol Chem (2017) 292(19):7866–87. doi: 10.1074/jbc.M116.768283 PMC5427267 28330872

[42] Iveson TJ Sobrero AF Yoshino T Souglakos I Ou FS Meyers JP. Duration of Adjuvant Doublet Chemotherapy (3 or 6 Months) in Patients With High-Risk Stage Ii Colorectal Cancer. J Clin Oncol (2021) 39(6):631–41. doi: 10.1200/jco.20.01330 PMC8078416 33439695

[43] Longley DB Harkin DP Johnston PG. 5-Fluorouracil: Mechanisms of Action and Clinical Strategies. Nat Rev Cancer (2003) 3(5):330–8. doi: 10.1038/nrc1074 12724731

[44] Zimmermann M Li T Semrad TJ Wu CY Yu A Cimino G. Oxaliplatin-DNA Adducts as Predictive Biomarkers of Folfox Response in Colorectal Cancer: A Potential Treatment Optimization Strategy. Mol Cancer Ther (2020) 19(4):1070–9. doi: 10.1158/1535-7163.Mct-19-0133 PMC7192311 32029633

[45] Chiu SJ Lee YJ Hsu TS Chen WS. Oxaliplatin-Induced Gamma-H2ax Activation Via Both P53-Dependent and -Independent Pathways But Is Not Associated With Cell Cycle Arrest in Human Colorectal Cancer Cells. Chem Biol Interact (2009) 182(2-3):173–82. doi: 10.1016/j.cbi.2009.08.019 19735649

[46] Attoub S Arafat K Khalaf T Sulaiman S Iratni R. Frondoside a Enhances the Anti-Cancer Effects of Oxaliplatin and 5-Fluorouracil on Colon Cancer Cells. Nutrients (2018) 10(5):1–12. doi: 10.3390/nu10050560 PMC5986440 29724012

[47] Xu K Chen Z Cui Y Qin C He Y Song X. Combined Olaparib and Oxaliplatin Inhibits Tumor Proliferation and Induces G2/M Arrest and Γ-H2ax Foci Formation in Colorectal Cancer. Onco Targets Ther (2015) 8:3047–54. doi: 10.2147/ott.S89154 PMC4622093 26543375

[48] Silva VR Corrêa RS Santos LS Soares MBP Batista AA Bezerra DP. A Ruthenium-Based 5-Fluorouracil Complex With Enhanced Cytotoxicity and Apoptosis Induction Action in Hct116 Cells. Sci Rep (2018) 8(1):288. doi: 10.1038/s41598-017-18639-6 29321581 PMC5762908

[49] Xie J Shults K Flye L Jiang F Head DR Briggs RC. Overexpression of Gsta2 Protects Against Cell Cycle Arrest and Apoptosis Induced by the DNA Inter-Strand Crosslinking Nitrogen Mustard, Mechlorethamine. J Cell Biochem (2005) 95(2):339–51. doi: 10.1002/jcb.20440 15778998

