# Supplementary material for: Glutathione S-Transferase Alpha 4 Promotes Proliferation and Chemoresistance in Colorectal Cancer Cells
Source: Front Oncol. 2022 Jul 1;12:887127. doi: 10.3389/fonc.2022.887127 (PMC9346510; doi:10.3389/fonc.2022.887127)
Supplement: Supplementary file 1 [file DataSheet_1.pdf]

Table 1. Guide RNAs and PCR primers used in this study

Gene/gRNA	sequence
gRNA66	
forward	CACCGCAGCAGGCGGCTCTAGCGCG
reverse	AAACCGCGCTAGAGCCGCCTGCTGC
gRNA214	
forward	CACCGCCCATCTCACGGACTCCATC
reverse	AAACGATGGAGTCCGTGAGATGGGC
hGSTA4-ND	
forward	GTCTCACTCTCGCATTCTTC
reverse	CCAATTTCGCGTCTTCAACC
hGSTA4	
forward	ATCAACCCTGGCGATCAATG
reverse	TGGTGGACGAGAACTAGAAC

Supplementary figure legends

Figure S1. Sequencing analysis confirms deletion of GSTA4 gene fragment. A 1135-bp fragment across exons 1 and 2 in GSTA4 gene is deleted.

Figure S2. Inactivation of GSTA4 has no effect on apoptosis. (A) Representative dot plots for FACS analysis of HCT116 and HCT116$^{\Delta GSTA4}$ cells exposed to PBS (Ctrl), 5-FU, and oxaliplatin, respectively. (B-D) No significant differences are noted in the proportions of early (B), late (C), and total (D) apoptotic cells between HCT116 and HCT116$^{\Delta GSTA4}$ cells exposed to PBS (Ctrl), 5-FU, and oxaliplatin, respectively (P > 0.05 for all comparisons). All data represent mean ± SEM from three independent experiments. ns, not significant.

Figure S3. FACS analysis for γH2AX following oxaliplatin treatment. (A) Representative histograms of FACS analysis for γH2AX. (B) No significantly increased proportion of γH2AX-positive cells is seen for HCT116$^{\Delta GSTA4}$ compared to HCT116 cells following oxaliplatin treatment. All data represent mean ± SEM for three independent experiments. ns, not significant.

Figure S4. Photographs of xenograft tumors. Five mice were enrolled in each group and tumor grew on both sides of flanks. In HCT116$^{\Delta GSTA4}$-xenografted group, one mouse treated with 5-FU and another mouse treated with oxaliplatin, died at early stage of experiment, respectively.

Figure S1

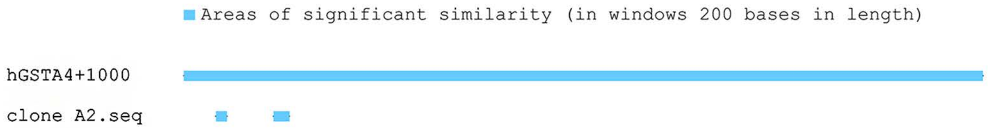


```
hGSTA4+1000    881 agcatagcaaatatcatttattcccaaaatgctaaagtttgagataaacggacttgatttccggctgttttgacactatccagaatgccttgcagatgggtggggcatgc
clone A2.seq   150 agcatagcaaatatcatttattcccaaaatgctaaagtttgagataaacggacttgatttccggctgttttgacactatccagaatgccttgcagatgggtggggcatgc

hGSTA4+1000    991 taaatactgcagtctccattggtgaggtcgtcccggagcctcgcccagctcccgcgcgctagagccgcctgctggtctcacccagccgggaccgctgacctggcgctttg
clone A2.seq   260 taaatactgcagtctccattggtgaggtcgtcccggagcctcgcccagctcccgcgc-----------------------------------------------------

hGSTA4+1000   1101 tgcggctccaggcctccgagtggactccaggtctgtctccctcctgcggtcgccccttcctcgccctttctctcttctttttttccctcccctttactctctgagtct
clone A2.seq       --------------------------------------------------------------------------------------------------------------

hGSTA4+1000   1211 cactctcgcattcttccctcactcccccagttcagtccttctcttgtcttttcttcctcttgtcctttgtcctcctgctccatccagtctgggcagaggtgggccccttg
clone A2.seq       --------------------------------------------------------------------------------------------------------------

hGSTA4+1000   1321 gctggagctggggtccttcttggctcagtgtgcgtatttttttttaaaccccttctgcggagtctgaatgcattggtggaaggtgggctcggatcgtccccgggcctggc
clone A2.seq       --------------------------------------------------------------------------------------------------------------

hGSTA4+1000   1431 tgggctgccgctacgcccctgcatgggctgagcggggaagacgagcatgggagagtgcgatctcagatggccaagccctggcccccggctcgttttacaaggccgggggc
clone A2.seq       --------------------------------------------------------------------------------------------------------------

hGSTA4+1000   1541 tcatctccctgtaggggtggtggacacgcgggacccgggggcgcagccaccgcatgcgtgacggtggacgcacattttgcagacagtagtagtactcggcctgttcacc
clone A2.seq       --------------------------------------------------------------------------------------------------------------

hGSTA4+1000   1651 ttccagttgccgtgataataaaatatgatgatgtatgtaaaagcatgaatacttgaagagcttccagtggctgtgcgattttccttcgctaccctcttcaacgcctgcca
clone A2.seq       --------------------------------------------------------------------------------------------------------------

hGSTA4+1000   1761 ccccctatcccccagctgaatttggaaaatctgagattctcgaggtcaaaatatataatattctgaatttgtgaaatcccgtactgctagcacatatgtttttacaacaa
clone A2.seq       --------------------------------------------------------------------------------------------------------------

hGSTA4+1000   1871 atggccccagcttcccaagatccaggataagaagtcataaatagaaatcatataaaagccaaggtttttttctccatgttttaaaatggtggtttttttttcttgagtga
clone A2.seq       --------------------------------------------------------------------------------------------------------------

hGSTA4+1000   1981 gggaaaatgcagcaacaaaaataaagaacagtttcccgtgccaaaatcctgaatattcatttcgcccctgagcactgggttgaagacgcgaaattgggactgttggtctg
clone A2.seq       --------------------------------------------------------------------------------------------------------------

hGSTA4+1000   2091 cgacgagctgctgcatttgtatttcagaaagcctgaaaagctatcatggcagcaaggcccaagctccactatcccaacggaagaggccggatggagtccgtgagatgggt
clone A2.seq   317 --------------------------------------------------------------------------------------------ggagtccgtgagatgggt

hGSTA4+1000   2201 tttagctgccgccggagtcgaggtaccttatatgctgtttcttcacggatttgtcatacagcttttgagacctttgaaagaaaaatacgttttgaagcaccgttcagttc
clone A2.seq   335 tttagctgccgccggagtcgaggtaccttatatgctgtttcttcacggatttgtcatacagcttttgagacctttgaaagaaaaatacgttttgaagcaccgttcagttc

hGSTA4+1000   2311 tagttctcgtccaccaaaatcacaggcacgcttcactgagtcagataccatccctgaagggatttttctttataattagaacgtagttgatggaattctattttcctttt
clone A2.seq   445 tagttctcgtccaccaaaatcacaggcacgcttcactgagtcagataccatccctgaagggatttttctttataattagaacgtagttgatggaattctattttcctttt
```

Figure S2

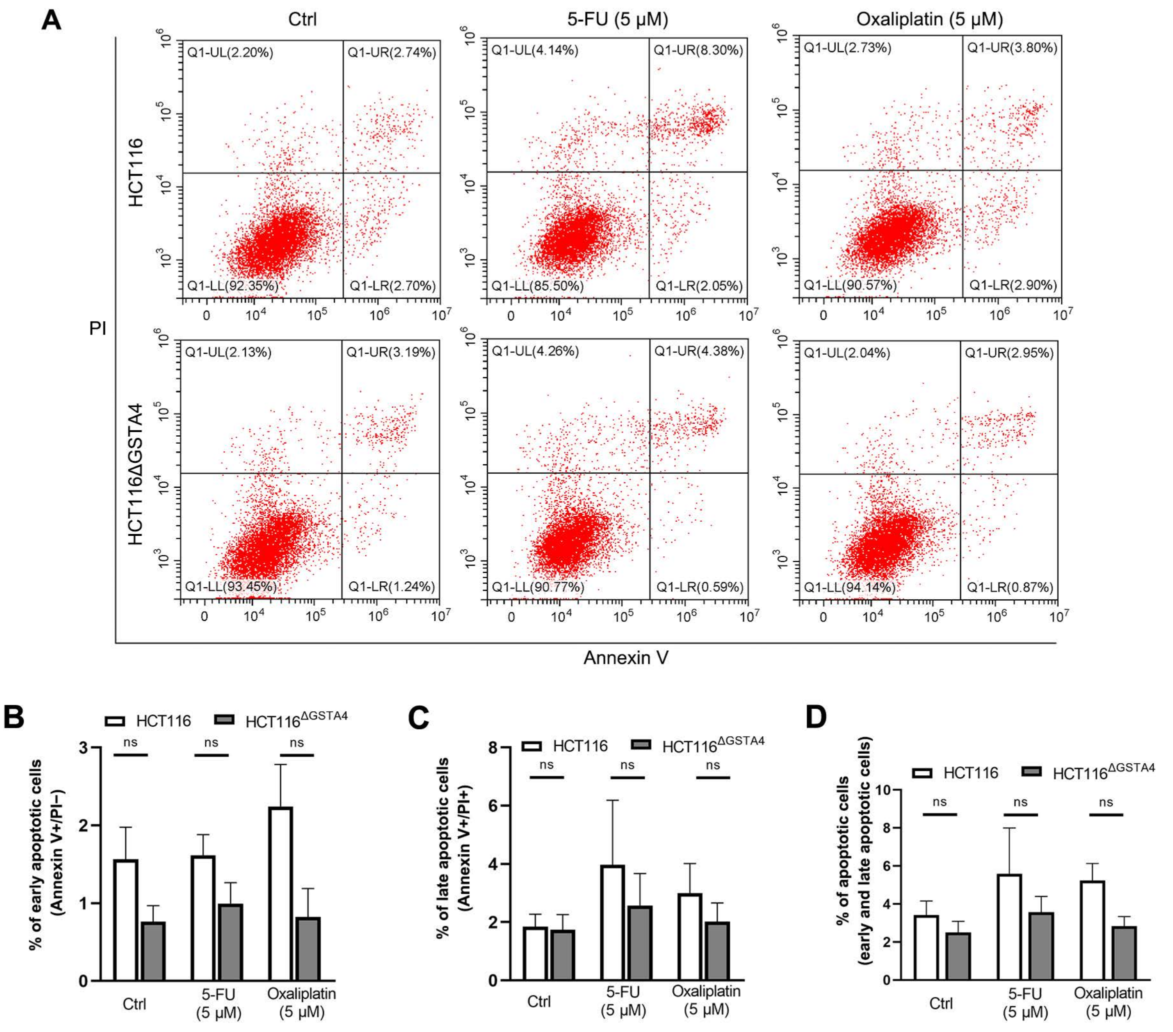
A
Ctrl
5-FU (5 μM)
Oxaliplatin (5 μM)
HCT116
HCT116ΔGSTA4
PI
Annexin V
Q1-UL(2.20%)
Q1-UR(2.74%)
Q1-LL(92.35%)
Q1-LR(2.70%)
Q1-UL(4.14%)
Q1-UR(8.30%)
Q1-LL(85.50%)
Q1-LR(2.05%)
Q1-UL(2.73%)
Q1-UR(3.80%)
Q1-LL(90.57%)
Q1-LR(2.90%)
Q1-UL(2.13%)
Q1-UR(3.19%)
Q1-LL(93.45%)
Q1-LR(1.24%)
Q1-UL(4.26%)
Q1-UR(4.38%)
Q1-LL(90.77%)
Q1-LR(0.59%)
Q1-UL(2.04%)
Q1-UR(2.95%)
Q1-LL(94.14%)
Q1-LR(0.87%)
B
HCT116
HCT116ΔGSTA4
% of early apoptotic cells (Annexin V+/PI−)
ns
Ctrl
5-FU (5 μM)
Oxaliplatin (5 μM)
C
% of late apoptotic cells (Annexin V+/PI+)
D
% of apoptotic cells (early and late apoptotic cells)

Figure S3

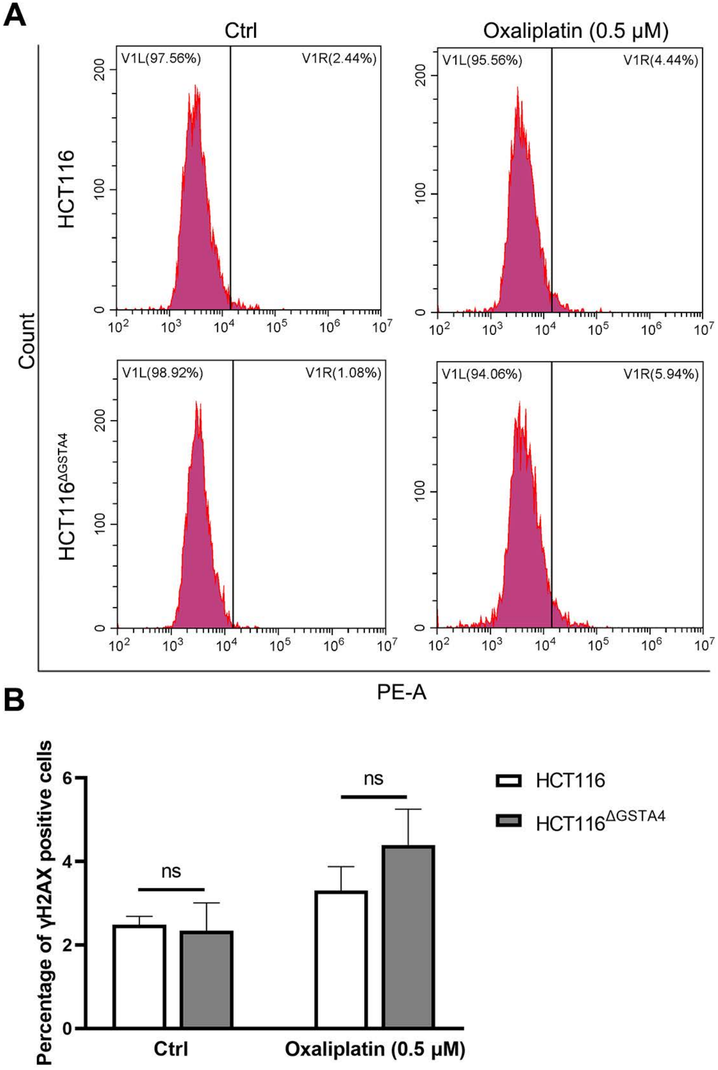

A
Ctrl
Oxaliplatin (0.5 μM)
V1L(97.56%)
V1R(2.44%)
V1L(95.56%)
V1R(4.44%)
V1L(98.92%)
V1R(1.08%)
V1L(94.06%)
V1R(5.94%)
HCT116
HCT116ΔGSTA4
Count
PE-A
B
Percentage of γH2AX positive cells
ns
ns
HCT116
HCT116ΔGSTA4
Ctrl
Oxaliplatin (0.5 μM)

Figure S4

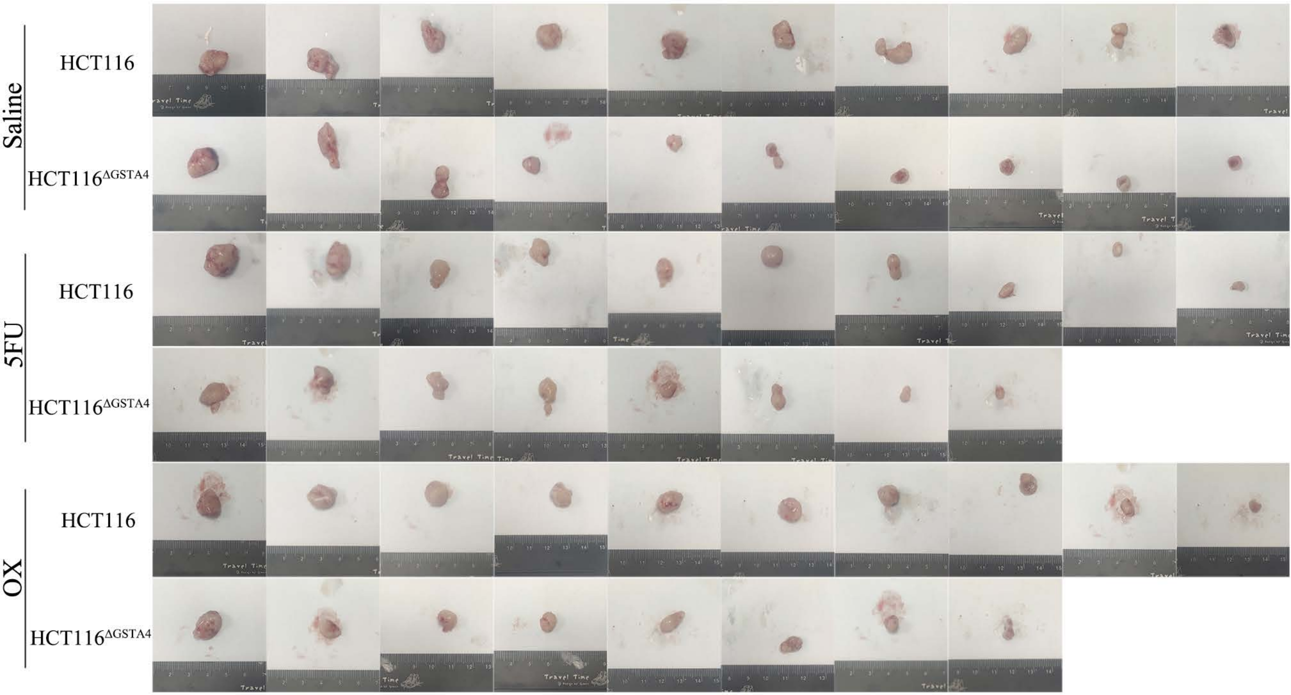
Saline
HCT116
HCT116$^{\Delta GSTA4}$
5FU
HCT116
HCT116$^{\Delta GSTA4}$
OX
HCT116
HCT116$^{\Delta GSTA4}$
